# Electrospun Fibers of Polybutylene Succinate/Graphene Oxide Composite for Syringe-Push Protein Absorption Membrane

**DOI:** 10.3390/polym13132042

**Published:** 2021-06-22

**Authors:** Nuankanya Sathirapongsasuti, Anuchan Panaksri, Sani Boonyagul, Somchai Chutipongtanate, Nuttapol Tanadchangsaeng

**Affiliations:** 1 Section of Translational Medicine, Faculty of Medicine Ramathibodi Hospital, Mahidol University, 270 Rama VI Rd., Thung Phaya Thai, Ratchathewi, Bangkok 10400, Thailand; nuankanya.sat@mahidol.edu; 2 Research Network of NANOTEC—MU Ramathibodi on Nanomedicine, Bangkok 10400, Thailand; 3 College of Biomedical Engineering, Rangsit University, 52/347 Phahonyothin Road, Lak-Hok 12000, Pathumthani, Thailand; anuchan.p59@rsu.ac.th (A.P.); sani@rsu.ac.th (S.B.); 4 Department of Pediatrics, Faculty of Medicine Ramathibodi Hospital, Mahidol University, 270 Rama VI Rd., Thung Phaya Thai, Ratchathewi, Bangkok 10400, Thailand; schuti.rama@gmail.com

**Keywords:** polybutylene succinate, filter membrane, electrospun fiber, graphene oxide, protein adsorption

## Abstract

The adsorption of proteins on membranes has been used for simple, low-cost, and minimal sample handling of large volume, low protein abundance liquid samples. Syringe-push membrane absorption (SPMA) is an innovative way to process bio-fluid samples by combining a medical syringe and protein-absorbable membrane, which makes SPMA a simple, rapid protein and proteomic analysis method. However, the membrane used for SPMA is only limited to commercially available protein-absorbable membrane options. To raise the method’s efficiency, higher protein binding capacity with a lower back pressure membrane is needed. In this research, we fabricated electrospun polybutylene succinate (PBS) membrane and compared it to electrospun polyvinylidene fluoride (PVDF). Rolling electrospinning (RE) and non-rolling electrospinning (NRE) were employed to synthesize polymer fibers, resulting in the different characteristics of mechanical and morphological properties. Adding graphene oxide (GO) composite does not affect their mechanical properties; however, electrospun PBS membrane can be applied as a filter membrane and has a higher pore area than electrospun PVDF membrane. Albumin solution filtration was performed using all the electrospun filter membranes by the SPMA technique to measure the protein capture efficiency and staining of the protein on the membranes, and these membranes were compared to the commercial filter membranes—PVDF, nitrocellulose, and Whatman no. 1. A combination of rolling electrospinning with graphene oxide composite and PBS resulted in two times more captured protein when compared to commercial membrane filtration and more than sixfold protein binding than non-composite polymer. The protein staining results further confirmed the enhancement of the protein binding property, showing more intense stained color in compositing polymer with GO.

## 1. Introduction

With the advances in protein and proteomics analysis technologies, the determination of protein in various bio-fluids has been extensively studied, especially in medical research, in which proteomic profiles or specific protein abundance were reviewed in many diseases [1,2,3,4,5]. In the environmental field, the determination of proteins in water samples could be an indicator of water quality and environmental risk factors [6,7]. Collecting samples from bio-fluids is, therefore, crucial for practical analysis, exclusively for extensive volume sampling. Syringe-push membrane absorption (SPMA) is an innovative way to reduce these problems. SPMA is a method for filtration of substances in liquid samples by filtration through a membrane which is pressurized by pressing the syringe. In this way, the suspended matter in the liquid is fixed on the membrane. Maintenance of the membrane of the sample reduces the sample volume stored for further examination [8]. However, the membrane must effectively capture the analytes or have a small pore size to filter the desired substance. Protein filtration currently uses a PVDF or nitrocellulose membrane to trap proteins [9]. However, the protein absorbance capacity is still low when compared to the original protein content in the fluid samples and the membrane easily tears when pressure is applied.

Therefore, this research aims to develop a membrane with the SPMA technique to have higher protein storage efficiency. The membrane was developed from polybutylene succinate (PBS) polymer, a polymer synthesized from natural materials. PBS is a polymer suitable for packaging, film, and textile applications, biodegrading in the environment [10]. PBS has high strength and flexibility, which is suitable for the pressure applied from the SPMA technique. From the previous study [11], an electrospinning method can be employed to produce a PBS fiber membrane that can be applied in filtration. Electrospinning is a versatile method for fabricating polymer micro- and nano-fibers as components in wound dressings, protective clothing, filtration, and reinforcement fibers in composites [12]. The membranes obtained by this technique increase the surface area and provide micrometer porosity [13]. Their high surface areas are the significant factor dictating their potential in filter membrane applications, which is useful for liquid filtration [14]. In addition, the electrospun PBS fiber membrane has also incorporated graphene oxide into the fabrication. Graphene oxide (GO) is a product obtained by oxidizing graphene. Data on studies of the interactions between graphene oxide and proteins are of interest. Zhang’s research [15] shows that graphene oxide can immobilize proteins, signaling that they are due to the chemical function of the two compounds. Graphene contains carboxyl groups, while proteins have amine groups, forming several bonds between chemical functions. This indicates that GO can bond with protein chemical groups, which may be useful for helping to trap proteins.

In this research, we fabricated electrospun PBS and a PBS/GO composite membrane, comparing it to electrospun PVDF, a material currently used with the SPMA technique, and PVDF/GO with the different fabrication methods. The two types of collector methods—rolling electrospinning (RE) and non-rolling electrospinning (NRE)—were employed to synthesize polymer fibers, resulting in different characteristics of mechanical and morphological properties. Moreover, all the electrospun filter membranes were performed during the filtration of the albumin solution by the SPMA technique to measure the protein capture efficiency as well as staining of the protein on the filter membrane, and were also compared with commercial filter membranes of PVDF, nitrocellulose and Whatman no. 1 paper.

## 2. Materials and Methods

### 2.1. Materials

Polybutylene succinate (PBS) (FD92) was supplied by PTT MCC Biochem Ltd (Bangkok, Thailand). Poly(vinylidene fluoride) (PVDF) was purchased from Sigma-Aldrich (batch no. 427144, St. Louis, MO, USA), with an average molecular weight of 275 kg/mol, according to the manufacturer. Graphene oxide powder, 15–20 sheets, 4–10% edge-oxidized, was purchased from Sigma-Aldrich (St. Louis, MO, USA). Dimethyl sulfoxide (DMSO) was purchased from Sigma-Aldrich (St. Louis, MO, USA). Acetone, methanol and chloroform were purchased from RCI Labscan (Bangkok, Thailand). Brilliant Blue Coomassie G-250 was purchased from ACROS Organics™. Hybridization Nitrocellulose Filter membrane (filter type—0.45 μm HATF) was purchased from Merck Millipore Ltd., Cork, Ireland. Transfer PVDF membrane (Immobion-P Transfer membrane, pore size 0.45 μm) was purchased from EMD Millipore Corporation, Burlington, MA, USA. Whatman no. 1 filter papers were purchased from GE Healthcare Life Sciences, Marlborough, MA, USA. Human albumin was purchased from Sigma-Aldrich, St. Louis, MO, USA.

### 2.2. Methods 

#### 2.2.1. Solution Preparation for Electrospun Fiber Composite Membrane

The 20 wt.% PVDF solution was prepared by dissolving PVDF in a mixture of DMSO and acetone (DMSO/acetone) 7:3 (volume ratio), with heating at 50 °C for 24 h. The 8 wt.% PBS solution was prepared in the same way as the PVDF, but the solvent was changed to chloroform/methanol 7:3 (volume ratio). The prepared polymer solution was divided into two conditions: with and without graphene oxide. A composite polymer solution was added with graphene oxide for 0.1% by weight of the polymer, mixed at a temperature of 60 °C, and then, subsequently underwent 15 min sonication.

#### 2.2.2. Fabrication of Electrospun Fiber Composite Membrane by Electrospinning

The prepared polymer solution was fabricated into a filter membrane using a single nozzle electrospinning setup with a flat grounded collector (non-rolling electrospinning, NRE) or rotating drum collector (rolling electrospinning, RE) with the speed set at 500 rpm. A high voltage power supply (ES60P-10 W, Gamma High Voltage, Ormond Beach, FL, USA) was attached to a metal nozzle to create a voltage difference between the metal nozzle and a collector of 25 kV. A charged metal nozzle with 0.57 mm outer diameter was placed 15 cm directly from the collector covered with aluminum foil. A mechanical pump (NE-300 Just Infusion™ Syringe Pump New Era Pump Systems, Inc., Farmingdale, NY, USA) was used to deliver the solution from a plastic syringe to the metal nozzle at a rate of 3 mL/h, with a feeding time of 4 h.

#### 2.2.3. Mechanical Properties Test and Uniformity Measurement of Membranes

A rectangular 1 × 2 cm^2^ membrane sample attached at both ends with 2 × 3 cm^2^ sandpaper was prepared. The mechanical properties of the electrospun and commercial membranes were evaluated using a universal test machine (TestResources, Shakopee, MN, USA) with a 5 kg load cell equipped with tensile grips. The stress–strain tests with a strain rate of 10 mm/min were performed until fracture occurred, and the data were obtained in an average of three measurements. Membrane size uniformity was measured from a standard deviation of membrane mean thickness. The membrane thickness was measured with a precise micrometer, randomly measuring five different points.

#### 2.2.4. Examination of Morphology by Scanning Electron Microscopy

A scanning electron microscope (SEM) (Prisma E, Thermo Scientific, Waltham, MA, USA) was used to characterize the electrospun fibers’ morphology and the size of the prepared 2 × 2 cm^2^ samples. Samples of the electrospun membranes were sputter-coated with gold. Images were captured with an accelerating voltage of 5 kV and were recorded at 16000× magnification. Image J software (www.imagej.nih.gov, Accessed on 1 May 2021) was used for measuring fiber diameter and pore size. The average fiber diameter and average pore area with standard deviation (SD) were determined from ten measurements.

#### 2.2.5. Comparison of Membrane Protein Binding Capacity

Three membranes of each condition were cut in a circular shape, 13 mm in diameter (n = 3). An amount of 1 mg/mL human albumin solution was prepared in deionized (DI) water. Then, 5 mL of prepared albumin solution was pushed through each filter membrane by the SPMA technique. The membrane adsorption capacity was relatively calculated from flowed through protein. An amount of 10 μL of each membrane flow-through fluid was used for protein measurement using a Bradford reagent kit (Bio-Rad), according to the manufacturer’s protocol. The flow-through protein concentrations of each filter membrane are shown in Figure S1. The albumin proteins attached to the electrospun filter (% protein adsorption) were calculated by Equation (1).

(1) $$\%\;Protein\;adsorption=\left(\frac{1-x}{1}\right)\times 100$$

where 1 is 100% albumin in the testing solution, and *x* is the amount of albumin that passed through the filter.

#### 2.2.6. Staining of Protein on the Filtered Specimens

Protein staining was prepared using a Neuhoff dye containing 2% H_3_PO_4_, 10% (NH_4_)_2_SO_4_, 20% methanol, and 0.1% Coomassie G-250, respectively. A total of 200 μL of dye solution was pipetted on the filter membrane that had already undergone the SPMA technique. After 30 min, the dye reagent was rinsed off with DI water. After that, the filter specimens were dried in the air, and then, photographs were taken for qualitative investigation of the protein on each filter membrane.

#### 2.2.7. Statistical Analysis

Data were analyzed using the SPSS Statistics software version 25.0 (IBM, Armonk, NY, USA) and Microsoft Excel 2019 (Microsoft, Redmond, WA, USA). A paired t-test was used for the comparison of two sets of data. The differences between means at the 95% confidence level (*p* < 0.05) were considered statistically significant.

## 3. Results and Discussion

### 3.1. Mechanical Properties and Membrane Thickness Uniformity

Table 1 shows the mechanical properties and the thickness of the various fabricated membrane conditions, depending on the fiber collection method and polymer types. The PBS and PVDF fibers via electrospinning were processed by high voltage on the needle and zero voltage on both collectors (flat grounded collector (non-rolling electrospinning, NRE) and rotating drum collector (rolling electrospinning, RE)). It can be noted that the PBS/solvent droplet can be pushed by high voltage on the needle to the collector; however, the droplet had no ability to form fibers on the flat collector. These may result from a solution jet that rapidly elongates the solution into micro- or nano-size until the solvent is completely evaporated, before the fiber drops on the collector. However, for rotating drum collectors, both PBS and PVDF could form fibers with the rolling electrospinning technique.

As shown in Table 1, the electrospun fiber membrane fabricated by NRE exhibits non-uniform thickness, while the RE membranes have uniformity. The uneven thickness of the NRE membrane indicates that the pattern in the membrane’s fabrication had low consistency due to the inability to direct the injected fibers to the plate collector. However, for rolling electrospinning (RE), the injected fibers could be controlled, to some extent, due to the presence of a rotating drum, which set the fiber direction to uniformity [16]. The electrospun membranes of all conditions have a substantially higher value of each mechanical property of Young’s modulus, maximum tensile strength, and elongation at break than commercial PVDF and nitrocellulose membranes, indicating more suitable strength for protein filtration. For all electrospun membranes, the addition of graphene oxide in PBS and PVDF polymers did not affect to the specimen’s mechanical behaviors, which were consistent with the study of PBS composited with graphene [17]. In the comparison of non-rolling electrospinning and rolling electrospinning, it was shown that the membranes from RE had a higher Young’s modulus than the NRE membranes. When applying pressure, the SPMA technique was typically used to filter proteins pressurized from the syringe through a membrane, where the membrane must be strong and flexible to support the force. Both polymers respond well to the above properties. It is indicated that both electrospun PBS and PVDF, with or without GO composite membranes, could tolerate the pushed pressure.

### 3.2. Morphology of the Fabricated Membranes

**Figure 1:**
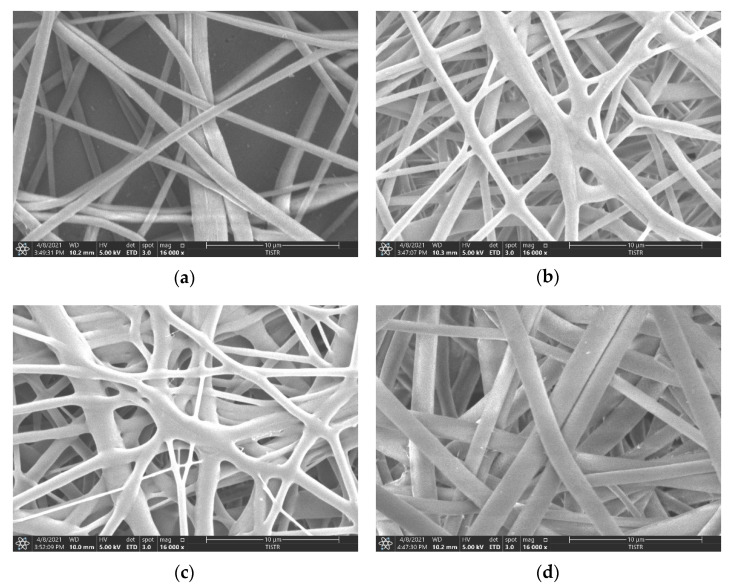

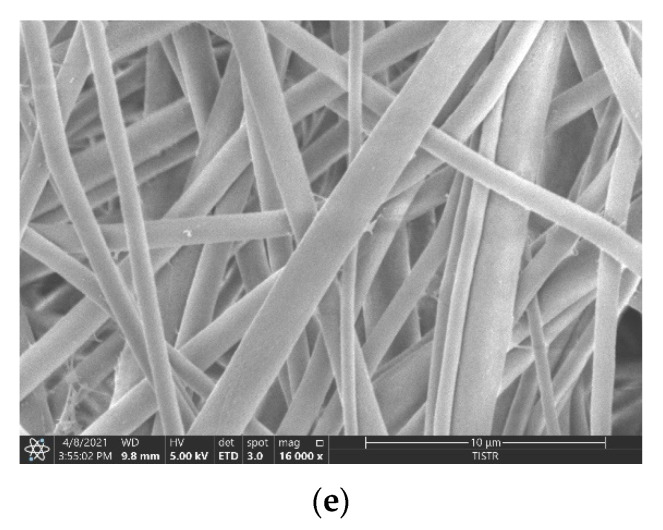
Morphology of electrospun fibers at 16,000× magnification. (**a**) PBS rolling electrospinning, (**b**) PBS + GO 0.1% rolling electrospinning, (**c**) PVDF non-rolling electrospinning, (**d**) PVDF + GO 0.1% non-rolling electrospinning, and (**e**) PVDF + GO 0.1% rolling electrospinning.

The images of the electrospun fibers of the membrane in each condition through a scanning electron microscope (SEM) are shown in Figure 1.

Figure 1 shows the characteristics of the fibers in 16,000× SEM images. The nature of the fibers formed by different methods with and without GO composite indicates the fiber overlap patterns and the size of the fiber gaps. However, we can detect the GO fragments trapped on the expanding fibers in the graphene oxide-containing membranes. The morphologies of the nanofibers obtained through RE and NRE of PBS and PVDF are the same (all fine, smooth, and cylindrical fibers), but the diameter changes. As listed in Table 2, the average diameters of the PBS rolling electrospinning, PBS + GO 0.1% rolling electrospinning, PVDF non-rolling electrospinning, PVDF + GO 0.1% non-rolling electrospinning, and PVDF + GO 0.1% rolling electrospinning were 1.0, 0.87, 0.9, 1.17 and 1.05 µm, respectively, indicating that each condition had a similar average diameter, with PBS + GO 0.1% membranes having the smallest fibers. However, from the pore area measured with ImageJ software, PBS membranes were slightly smaller than PVDF membranes. These results indicate that the electrospun PBS membrane has a higher ability to be employed as a filter membrane than the electrospun PVDF membrane.

Membrane morphology substantially confirmed the hypothesis of the previously discussed mechanical properties. The morphology also suggests that variations can occur with rolling electrospinning, possibly depending on the polymer used in fabrication. Morphological results suggest that the fiber size and the porosity for each condition were similar to the previous research, with a fiber size of approximately 1 μm [18]. Commercial membranes (nitrocellulose membranes and PVDF membranes) have a smaller pore size than electrospinning membranes of PBS or PBS+GO. However, the electrospun membranes have an advantage over the contact surface area [19], which could increase protein adsorption ability. The morphology of PBS + GO 0.1% (RE) combined branched-flat and straight-round fibers, while that of PVDF + GO 0.1% (RE) contained only straight-flat and straight-round fibers. These phenomena may result in a higher area that can adsorb the small molecules of protein, giving rise to an increase in protein binding capacity of PBS + GO 0.1% (RE).

### 3.3. The Protein Binding Capacity of the Fabricated Filter Membranes and Commercially Available Filter Membranes

**Figure 2:**
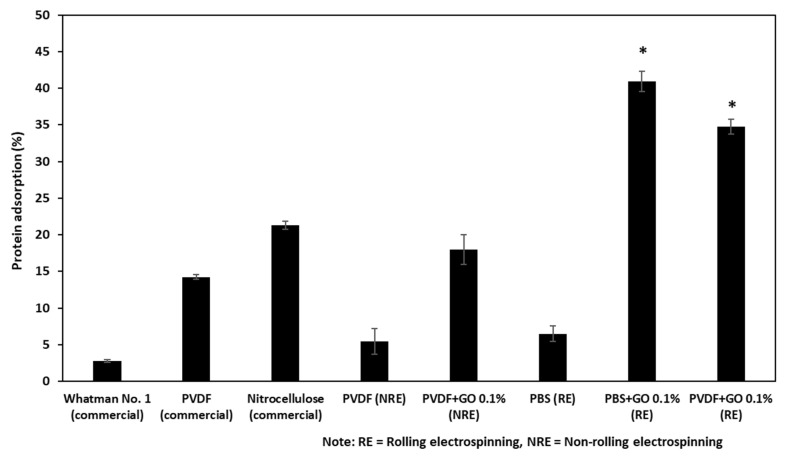
The protein adsorption capacity of five fabricated membranes and three commercial membranes used in this study. Note: * Significant differences of the two samples compared to the others.

The protein binding capacity of the fabricated filter membranes and commercially available filter membranes were compared based on a relative calculation from remnant protein in the flow-through fluid, as shown in Figure 2. The protein binding capacity was calculated using Equation (1) (details in Materials and Methods). Five different fabricated conditions and three commercial filter membranes were used for comparison of protein binding capacity. The experiments were performed in triplicate for each membrane. The results showed that Whatman no. 1 filter paper had the lowest protein binding capacity, followed by pure PVDF (NRE), and pure PBS (RE) with protein binding capacity of 2.76%, 5.48%, and 6.47%, respectively. The nitrocellulose showed the highest protein binding capacity among commercially available filter membranes, with a binding capacity of 21.32%. Using the same polymer, the pure PVDF filter membrane developed in this study showed less protein absorbance when compared with the commercial one, 5.48% and 14.24%; this might be because of the pore size, 0.90 ± 0.29 μm and 0.45 μm, and the random pattern of the electrospinning.

Interestingly, the addition of GO could improve the protein binding capacity dramatically to 3.29-fold when using non-rolling electrospinning and even higher to 6.5-fold with the rolling electrospinning. The results of PBS also showed a similar trend; adding GO coupled with the rolling electrospinning process could increase protein adsorption to 6.32-fold when compared with PBS without GO using the same manufacturing process. Taking all the results together, the PBS with GO 0.1% rolling electrospinning showed the highest protein capacity, about 2-fold higher than commercial nitrocellulose membrane and 2.88-fold higher when compared to the commercial PVDF membrane.

### 3.4. Qualitative Investigation of Absorbed Protein on the Membrane by Protein Staining

**Figure 3:**
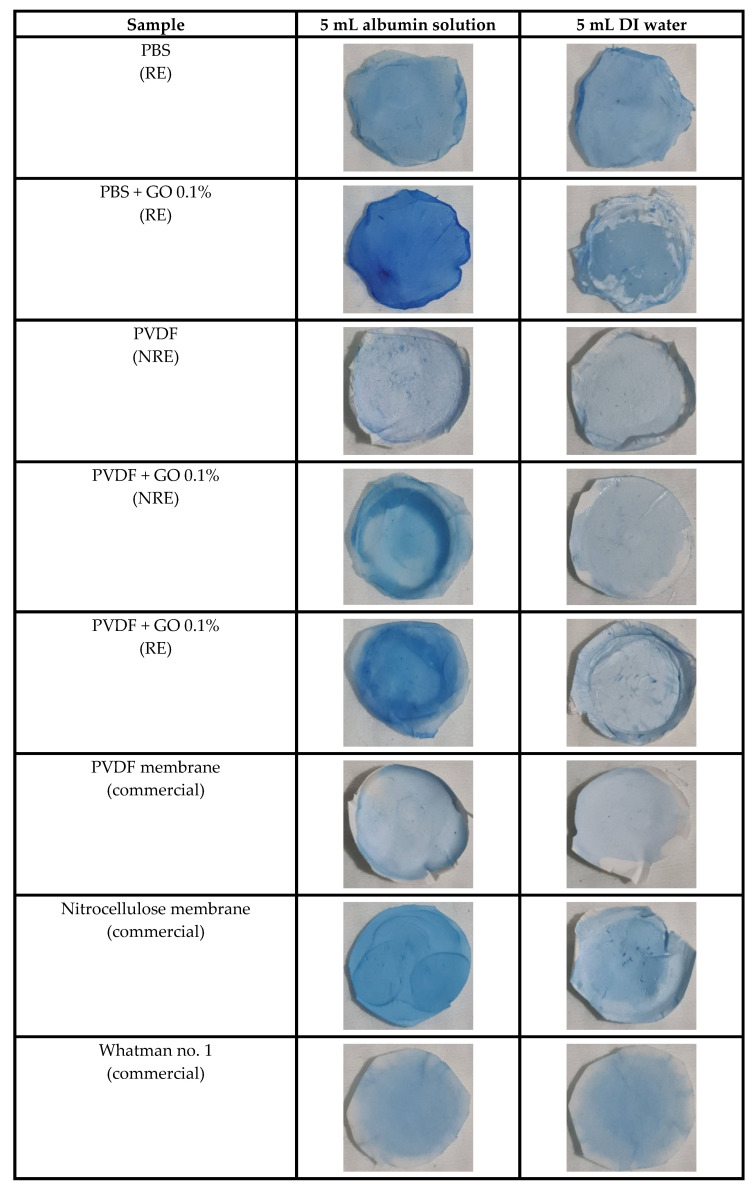
Brilliant blue Coomassie G-250 staining of absorbed proteins on membranes.

Protein staining with Neuhoff dye was applied to determine the distribution and absorbed protein on each filtered membrane. The blue color on each sample indicates the protein’s attachment on the membrane. The results of dyeing are shown in Figure 3. DI water filtration was used for baseline staining of the materials (blank control).

The results showed that PBS + GO 0.1% rolling electrospinning, PVDF + GO 0.1% rolling electrospinning, PVDF + GO 0.1% non-rolling electrospinning, and nitrocellulose had deep blue staining after albumin adsorption as compared to fainter blue staining after DI water adsorption. Interestingly, the graphene oxide-based membranes showed more intense dye staining than that of non-graphene-added samples. Commercial membranes (PVDF and nitrocellulose) exhibited lighter blue staining than those containing graphene oxide. Moreover, the fabricated procedure, rolling electrospinning, and non-rolling electrospinning resulted in different adsorption capacities. The Whatman no. 1 filter paper, as the negative control, presented very light blue staining in both protein and blank control filtering, indicating very low protein adsorption ability. The PBS + GO 0.1% still has the highest value of color intensity, compared to the other intensities of the protein staining data, as shown in Figure S2. These quantitative intensity results are consistent with the data of the protein adsorption experiment. These results indicate that the membrane morphology also supported the reasoning in terms of fabrication patterns and three-dimensional structure that increase surface area and aid in protein capture [20].

## 4. Conclusions

Syringe-push membrane absorption (SPMA) is a rapid and straightforward method of bio-fluid preparation for protein and proteomic study. However, the commercially available membranes still have some drawbacks: low protein adsorption capacity and tearing when pressure is applied. This research, employing PBS composite with GO and fabricating electrospun membrane on the rolling electrospinning, resulted in the best performance and could solve previous SPMA issues. Further scaled-up production, protein recovery optimization, and recovered protein yield evaluation would be crucial steps toward practical implementation. To the best of our knowledge, this research is the first experiment that reports the utilization of the electrospun bioplastic as a protein adsorption membrane for further biomedical applications.

## Figures and Tables

**Table 1:** Sample thickness and mechanical properties of each filter membrane used in this study.

Sample	Thickness (μm)	Young’s Modulus (MPa)	Max. Tensile Strength (MPa)	Elongation at Break (%)
PBS (RE)	30.8 ± 7.9	193 ± 3.0	24 ± 3.6	58 ± 6.5
PBS + GO 0.1% (RE)	45.2 ± 6.9	170 ± 2.0	31 ± 4.6	50 ± 2.6
PVDF (NRE)	116.8 ± 47.3	143 ± 5.6	18 ± 3.6	61 ± 7.2
PVDF + GO 0.1% (NRE)	87.6 ± 47.3	101 ± 2.6	24 ± 2.0	53 ± 5.2
PVDF + GO 0.1% (RE)	82.4 ± 3.2	181 ± 2.0	32 ± 4.0	57 ± 5.2
Nitrocellulose (commercial)	140.4 ± 1.0	4.8 ± 0.6	0.18 ± 0.1	9 ± 2.6
PVDF (commercial)	120.2 ± 1.0	5.3 ± 1.2	0.12 ± 0.1	9 ± 3.5

Note: RE is rolling electrospinning, NRE is non-rolling electrospinning.

**Table 2:** Fiber diameter and membrane area from SEM images.

Sample	Average Diameter of Fibers (μm)	Average Pore Area (μm^2^)
PBS (RE)	1.00 ± 0.27	27.01 ± 4.48
PBS + GO 0.1% (RE)	0.87 ± 0.16	23.32 ± 2.98
PVDF (NRE)	0.90 ± 0.29	36.26 ± 5.86
PVDF + GO 0.1% (NRE)	1.17 ± 0.22	37.48 ± 6.25
PVDF + GO 0.1% (RE)	1.05 ± 0.22	33.29 ± 8.33

## Data Availability

The data presented in this study are available on request from the corresponding author.

## Supplementary Materials

The following are available online at https://www.mdpi.com/article/10.3390/polym13132042/s1, Figure S1: Calibration curve of Protein vs. OD. Figure S2: Color intensities of the staining membranes.
