# Supplementary material for: Electrospun Fibers of Polybutylene Succinate/Graphene Oxide Composite for Syringe-Push Protein Absorption Membrane
Source: Polymers (Basel). 2021 Jun 22;13(13):2042. doi: 10.3390/polym13132042 (PMC8271884; doi:10.3390/polym13132042)

Supplementary Materials

Electrospun Fibers of Polybutylene Succinate/Graphene Oxide Composite for Syringe-push Protein Absorption Membrane

Nuankanya Sathirapongsasuti [1,2], Anuchan Panaksri [2,3], Sani Boonyagul [3], Somchai Chutipongtanate [4] and Nuttapol Tanadchangsaeng [3,*]

[1] Section of Translational Medicine, Faculty of Medicine Ramathibodi Hospital, Mahidol University, 270 Thanon Rama VI, Thung Phaya Thai, Ratchathewi, Bangkok 10400 Thailand; nuankanya.sat@mahidol.ac.th (N.S.)

[2] Research Network of NANOTEC - MU Ramathibodi on Nanomedicine, Bangkok, Thailand; nuankanya.sat@mahidol.ac.th (N.S.); anuchan.p59@rsu.ac.th (A.P.)

[3] College of Biomedical Engineering, Rangsit University, 52/347 Phahonyothin Road, Lak-Hok, Pathumthani 12000 Thailand; anuchan.p59@rsu.ac.th (A.P.); sani@rsu.ac.th (S.B.); nuttapol.t@rsu.ac.th (N.T.)

[4] Department of Pediatrics, Faculty of Medicine Ramathibodi Hospital, Mahidol University, 270 Thanon Rama VI, Thung Phaya Thai, Ratchathewi, Bangkok 10400 Thailand; schuti.rama@gmail.com (S.C.)

* Correspondence: nuttapol.t@rsu.ac.th; Tel.: +66-(0)2-997-2200 ext. 1428, Fax: +66-(0)2-997-2200 ext. 1408

Figure S1 Calibration curve of Protein vs. OD

Protein concentration (mg/ml)	OD. 595 nm (Abs.)
0.2	1.137
0.4	1.468
0.6	1.658
0.8	1.802
1	2.005

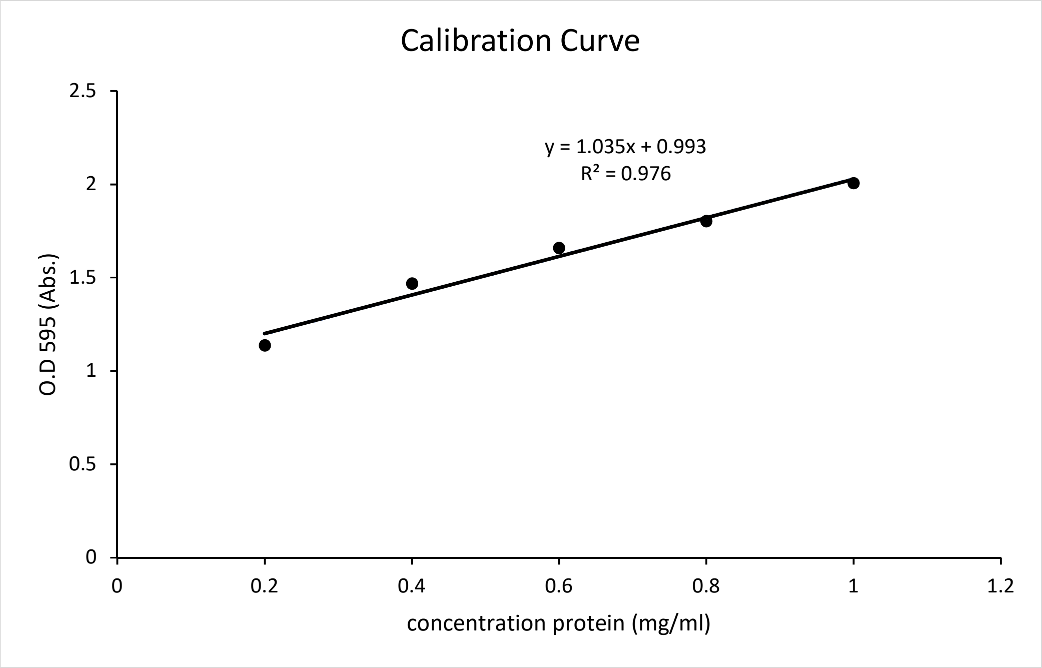

Figure S2 Color intensities of the staining membranes.

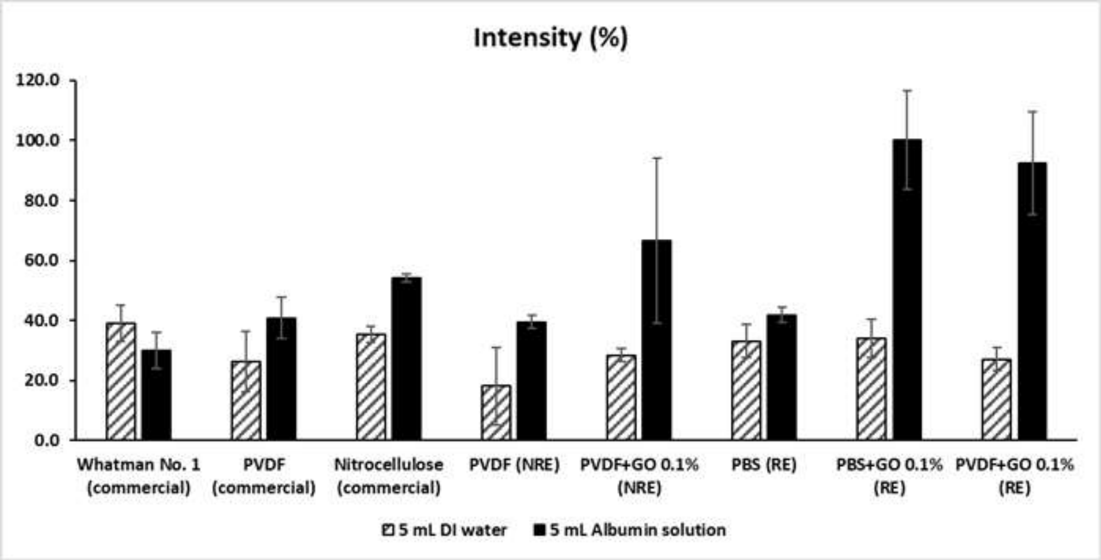

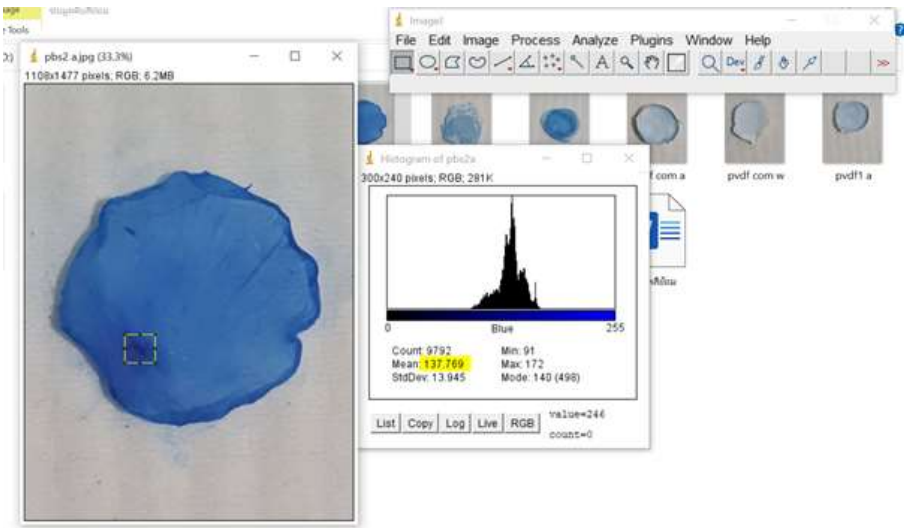

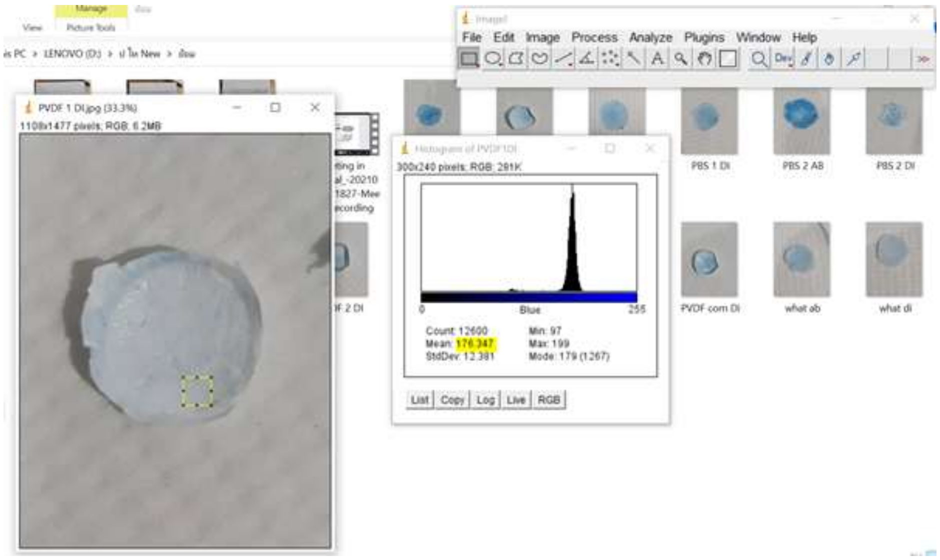

Supplement: Supplementary file 1 [file polymers-13-02042-s001.zip › polymers-1255904-supplementary.pdf]
